# Dietary Inflammatory Index (DII)^®^ and Metabolic Syndrome in the Selected Population of Polish Adults: Results of the PURE Poland Sub-Study

**DOI:** 10.3390/ijerph20021056

**Published:** 2023-01-06

**Authors:** Alicja Szypowska, Katarzyna Zatońska, Andrzej Szuba, Bożena Regulska-Ilow

**Affiliations:** 1 Department of Dietetics, Wroclaw Medical University, 50-556 Wroclaw, Poland; 2 Department of Population Health, Wroclaw Medical University, 50-368 Wroclaw, Poland; 3 Department of Angiology, Hypertension and Diabetology, Wroclaw Medical University, 50-556 Wroclaw, Poland; 4 Department of Dietetics and Bromatology, Wroclaw Medical University, 50-556 Wroclaw, Poland

**Keywords:** Dietary Inflammatory Index, metabolic syndrome, inflammation, nutrition, PURE study

## Abstract

The aim of the study was to assess the relationship between the inflammatory potential of the diets of residents of Lower Silesia, based on the Dietary Inflammatory Index (DII), with the incidence of metabolic syndrome (MetS) and its components. Diets were characterized according to DII terciles. The study group consisted of 1570 individuals enrolled in the Polish arm of the Prospective Urban and Rural Epidemiological (PURE) study. Participants’ diets in DII T1 (most anti-inflammatory diet) had the highest intake of vegetables (except for potatoes), fruits, nuts and seeds, low-calorie beverages, tea, and coffee (all *p* < 0.001). On the other hand, participants’ diets in DII T3 (most pro-inflammatory diet) contained a lot of whole-fat products, refined cereals, fats (except for vegetable oils), fruit juices, red meat, processed meat/meat products, sugar-sweetened beverages, sweets, sugar, and honey (all *p* < 0.001). Overall, we did not find an increased prevalence of MetS and its individual components in DII tercile 3 (T3) compared to DII tercile 1 (T1), except for an increased prevalence of abnormal TG in DII T3 compared to T1 (OR 1.34; 95% CI = 1.01 to 1.78) in the crude model. In the adjusted model, a lower prevalence of abnormal fasting glucose (FG) was found in DII T2 compared to DII T1 (OR 0.71; 95% CI = 00.54 to 0.94). Results of this study are informative and provide an important basis for further research on the quality of diet and nutrition.

## 1. Introduction

Metabolic syndrome (MetS) is a major health hazard of modern world [1]. According to the most current criteria accepted in 2009 by the International Diabetes Federation (IDF), the American Heart Association and National Heart, Lung and Blood Institute (AHA/NLBI) diagnose MetS if three of the following five features are found: elevated blood pressure (BP), elevated fasting glucose (FG), elevated triglyceride (TG), abdominal obesity, and reduced high-density lipoprotein cholesterol (HDL-C) [2]. Metabolic syndrome is associated with the development of cardiovascular disease, diabetes, non-alcoholic fatty liver disease, chronic kidney disease, some cancers, and even increased mortality [1,3,4,5]. Patients with MetS are at twice the risk of developing cardiovascular disease (CVD) over the next 5–10 years and a 5-fold increase in risk for type 2 diabetes mellitus [2].

There are many factors and mechanisms in the development of MetS, including insulin resistance, adipose tissue dysfunction, chronic inflammation, oxidative stress, abnormal microbiota, and genetics [2,6]. Chronic inflammation is associated with insulin resistance and visceral obesity. These factors are linked to the secretion of pro-inflammatory cytokines interleukin-1 (IL-1), IL-6, tumor necrosis factor alpha (TNF-α), adiponectin, and leptin [7,8]. This may be caused by a high-energy diet and cell death that induces local inflammation [6,9]. Inadequate dietary patterns have been linked to all metabolic disorders in MetS, and all of MetS individual components are modifiable risk factors for the development of CVD, meaning that making appropriate lifestyle changes can reduce the risk of their occurrence [10,11].

Dietary patterns rich in fruits, vegetables, whole grains, nuts, legumes, fish, olive oil and minimally processed foods, i.e., the Mediterranean and Dietary Approaches to Stop Hypertension (DASH) diets, are associated with the lowering of systemic inflammation and lower C-Reactive Protein (CRP) compared to unhealthy dietary patterns [12,13,14]. On the other hand, the consumption of a Western diet, rich in processed foods, simple carbohydrates, refined grains, red processed meat, saturated fatty acids, and sodium, leads to chronic inflammation and increased CVDs markers [15,16].

The Dietary Inflammatory Index (DII) is a scoring algorithm to classify individuals’ diets according to their inflammatory potential [17]. The authors of DII evaluated the association of dietary components with six inflammatory markers: IL-1β, IL-4, IL-6, IL-10, TNF-α, and CRP [17]. Currently, there is an increasing number of studies on the association between DII scores and the prevalence of MetS, but the obtained results are inconsistent [18,19,20,21,22,23]. A 2021 umbrella review showed that anti-inflammatory dietary patterns play a significant role in the prevention of chronic diseases [24]. However, studies on MetS and DII were identified as having no evidence (Class V) with no statistical significance using a *p*-value of > 0.05, except for MetS individual components whose associations were identified as suggestive (for waist circumference, WC), or weak (for systolic BP and FG). 

There are few studies that assessed dietary patterns with DII among the Polish population. Sokol et al. [18] examined the association between the DII and some MetS components among the Polish population from a specific geographic region (Świętokrzyskie Province and the city of Kielce). They found that mean waist-to-hip ratio (WHR) and diastolic blood pressure were greater among those in DII quartile 4 compared to 1 [18]. Our study evaluates the Polish population from Poland’s Lower Silesia Province and the city of Wroclaw. The prevalence of MetS among residents of Wroclaw has been evaluated previously [25]. In a randomized study by Ilow et al. [25] (*n* = 18,583) MetS occurred in 28.5% of the study group and its prevalence increased with age.

The aim of the study was to evaluate the relationship between inflammatory potential of the diets of urban and rural residents of Lower Silesia based on DII with the incidence of MetS and its components. Diets were characterized according to DII terciles (T).

## 2. Materials and Methods

### 2.1. Study Population

The study group consisted of 1570 individuals enrolled in the Prospective Urban and Rural Epidemiological (PURE) Poland sub-study. The inclusion criteria for the study were: aged 35–70 and permanent residence in urban or rural area of the Lower Silesia region. Individuals were recruited to the Polish arm of the PURE study through the radio and television announcements. The aim of the study was to calculate the association between urbanization and CVD prevalence. The main results of the study have been previously published [26,27]. The first stage of the study was conducted between 2007 and 2009, and it included a Food Frequency Questionnaire (FFQ), blood draws, BP measurements, spirometry, and anthropometric measurements. There were a total of 2039 study participants. Individuals who did not meet the criterion of adequate dietary energy intake (for men <800 kcal, >4200 kcal, and for women <600 kcal, >3500 kcal) were excluded. The study inclusion criteria were established in accordance with recommendations [28]. Participants were excluded from the study due to missing data for more than one variable (*n* = 221). Finally, a total of 1570 individuals were included in the study.

### 2.2. Data Collection

The concentrations of FG, TG, and HDL-C were measured from venous blood samples. A SPINREACT enzymatic test kit (Sant Esteve De Bas, Girona, Spain) was used to measure HDL-C and TG concentrations. FG was measured after an overnight fasting period with the Ascensia ENTRUST Glucometer kit (Bayer, Germany). The above variables were expressed in mmol/L. Systolic and diastolic BP was measured with a certified automatic BP monitor (Omron HEM-711 IntelliSense, Tokyo, Japan) and expressed in mm Hg. Study participants were recommended to rest for 5 min before BP measurement. 

In the PURE study, BP was measured twice. Waist circumference was measured once, midway between the lowest rib and the upper iliac crest, with a standard measuring tape to the nearest 0.5 cm.

### 2.3. Definition of the Metabolic Syndrome

The definition of MetS as declared by the IDF, and AHA/NLBI harmonized criteria for MetS [2], was used for MetS diagnosis, comprising of the presence of three or more of the following components:

- FG ≥ 5.6 mmol/L (100 mg/dL), or drug treatment for elevated glucose;
- Systolic BP ≥ 130 mm Hg, or diastolic BP ≥ 85 mm Hg, or antihypertensive drug treatment for previously diagnosed hypertension;
- HDL-C < 1.0 mmol/L(40 mg/dL) in males and <1.3 mmol/L (50 mg/dL) in women, or a history of drug treatment for this abnormality;
- WC ≥ 80 cm in women and ≥94 cm in men [2].

### 2.4. Dietary Intake Assessment

Participants’ habitual food intake was assessed with the country- and culture-specific FFQ, which was developed and validated for the population of PURE study from Lower Silesia [29]. The FFQ asked about the average consumption of food products during the year preceding the survey and assessed the frequency of consumption of 154 products, which were divided into 26 food groups (Table 1) [30]. The frequency of consumption of selected foods was assessed with 10 possible responses: never, less than once a month, 1–3 times a month, once a week, 2–4 times a week, 5–6 times a week, once a day, 2–3 times a day, 4–5 times a day, and >6 times a day. The nutritional value of the diets was calculated based on the Polish Food Composition Tables [31]. “The Album of photographs of food products and dishes” developed by the National Food and Nutrition Institute in Warsaw was used to determine the average size of the consumed portion [32]. The FFQ has been published previously [33]. The place of residence was classified as rural or urban, and education as elementary/unknown, trade, secondary/high school, or university. The International Physical Activity Questionnaire (IPAQ) was used to calculate physical activity and expressed as metabolic equivalent (MET) minutes per week. The number of MET-min/week lower than 600 was considered low, 600–3000 as moderate, and above 3000 as high [34]. Smoking status was classified into 3 categories: non-smoker, ex-smoker, and current smoker.

### 2.5. Dietary Inflammatory Index (DII)^®^ Calculation

DII is a scoring algorithm to classify individuals’ diets according to their inflammatory potential. A modified and updated version of DII developed by Shivappa et al. [17] was used in this study. Its detailed description has been described in Shivappa et al.’s [17] publication. Authors of DII calculated the global daily intake of individual dietary food components/food products, along with the standard deviation, based on 11 data sets from around the world (USA, Australia, the Kingdom of Bahrain, Denmark, India, Japan, New Zealand, Taiwan, South Korea, Mexico, and the UK). Dietary intake of the DII components by study participants was compared to the standard global as a Z-score, which was achieved by subtracting the standard mean from the amount reported and dividing this value by its standard deviation [17]. Then, this value was converted to a centered percentile score. To achieve a symmetrical distribution with values centered on 0 (null) and bounded between −1 (maximally anti-inflammatory) and +1 (maximally pro-inflammatory), each percentile score was doubled and then ‘1’ was subtracted. The centered percentile values were then multiplied by the overall pro- and anti-inflammatory effect score for each dietary component. Finally, all results were summed up. Higher DII scores indicated that the diet was more pro-inflammatory, and lower DII scores represented a more anti-inflammatory diet. Thirty-seven dietary components and food products were used to calculate the DII score, including 29 anti-inflammatory elements; monounsaturated fatty acids, polyunsaturated fatty acids, n-3 fatty acids, n-6 fatty acids, fiber, alcohol, vitamins A, D, E, C, and B_6_, β-carotene, thiamine, riboflavin, niacin, folic acid, magnesium, selenium, zinc, flavan-3-ol, flavones, flavonols, flavonones, anthocyanidins, isoflavones, caffeine, garlic, onion, and green/black tea, and 8 pro-inflammatory elements; carbohydrates, protein, total fat, saturated fatty acids, trans fat, cholesterol, iron, and vitamin B_12_. Energy-adjusted values (the nutrient density method) were used to decrease the influence of different energy intake among study participants [35].

### 2.6. Statistical Analysis

Nominal variables are presented as *n* (% of group), and continuous variables as mean ± SD or median (T1; T3). Normality of distribution in subgroups was evaluated using the Kolmogorov-Smirnov test, skewness, and kurtosis values, and were based on visual assessment of histograms. Comparison of diet parameters between DII terciles groups was made using one-way ANOVA or Kruskal-Wallis test (as appropriate). A post-hoc test (Tukey test for ANOVA and Dunn test for Kruskal-Wallis test) was used with Bonferroni correction for multiple comparisons. Additionally, logistic regression analysis was used to determine odds ratios (ORs) with 95% confidence intervals (CIs) for metabolic syndrome and its components according to the DII terciles. The bottom DII tercile (T1) was used as a reference category. We created both univariate and multivariable models. Multivariate models included age, sex, place of residence, education level, physical activity level, smoking status, and body mass index (BMI) as potential confounders. All tests were two-tailed with a significance level of 0.05. Statistical analysis was conducted using R software (a language and environment for statistical computing, v. 3.5.1. R Foundation for Statistical Computing, Vienna, Austria).

### 2.7. Ethical Approval

The study has been approved by the Polish Ethics Committee (No. KB-443/2006). All participants were volunteers and signed an informed consent form prior to all examinations. All participants were examined according to the global PURE [36] study protocol.

## 3. Results

Table 2 presents characteristics of the study group (*n* = 1570); socio-demographic, anthropometric, lifestyle-related data (smoking status, physical activity), prevalence of MetS and its components, as well as DII scores of participants’ diets. More than half of the study participants were women (*n* = 1000). The mean age of the study group was 54.65 ± 9.83. The mean BMI was 28.17 ± 5.15. Most of participants (74.3%) were married or cohabiting. More than half of the study participants had a high school education or less (70.8%) and reported smoking cigarettes currently or in the past, with men being more likely to smoke (64.8% vs. 55.3%). Most of Lower Silesia inhabitants (79.2%) declared current or previous alcohol consumption. The mean DII score in the study group was −0.11 ± 2.91. A significantly lower DII score was found in the group of women compared to the group of men (−0.61 ± 2.88 vs 0.77 ± 2.75, *p* < 0.001). The minimum DII score was −7.88, and the maximum DII score was 7.33. Metabolic syndrome was diagnosed in 42.3% of the study participants. The prevalence of MetS and its components was as follows: MetS (42.3%); BP (77.6%); WC (69.7%); HDL-C (19.7%); TG (24.6%); and FG (39.1%). Metabolic syndrome and abnormal levels of BP and TG were significantly more frequent in the group of men compared to the group of women (46.1% vs. 40.1%; 88.4% vs. 71.4%; 30.7% vs. 1.2%, respectively). Abnormal HDL-C levels were significantly more frequent in the group of women compared to the group of men (23.4% vs 13.3%). Other MetS components were not statistically significant.

Table 3 presents detailed characteristics of participants’ diets according to the DII terciles. Diets were assessed by calculating DII scores and then divided into terciles. The most anti-inflammatory diet (T1) ranged from −7.88 to −1.53, the moderate diet (T2) from −1.52 to 1.21, and the most proinflammatory diet (T3) from 1.22 to 7.33. Participants’ diets in T1 had the highest intake of vegetables (except for potatoes), fruits, nuts and seeds, low-calorie beverages, tea, and coffee. Pro-inflammatory diets (T3) had the highest intake of whole-fat foods, refined cereals, fats except for vegetable oils (butter, lard, fat spread, mayonnaise, soft margarine), fruit juices, red meat, processed meat, sugar-sweetened beverages, sweets, sugar, and honey. Moderate and pro-inflammatory diets (T2 and T3) had a similar intake of potatoes, breaded meat, boiled and fried eggs, mixed dishes, and soups. Fish intake was similar in T1 and T2. The remaining food components were not statistically significant.

Table 4 presents logistic regressions of MetS and its components according to the DII tertiles. Age, sex, place of living, education, physical activity, smoking status, and BMI were excluded as confounders. Overall, in the crude model we did not find an increased prevalence of MetS and its components in T3 compared to T1, except for increased TG levels (OR 1.34; 95% CI = 1.01 to 1.78). In the adjusted model, we found a lower prevalence of FG in T2 compared to T1 (OR 0.71; 95% CI = 00.54 to 0.94).

## 4. Discussion

We found no significant association between the inflammatory potential of the diet assessed by the DII and the risk of MetS. In this study, the prevalence of MetS was higher (i.e., 42.3% overall, 40.1% among women, and 42.3% among men) than in other studies conducted in the Polish population [18,25], which may be related to adopting different criteria. Our study adopted the IDF definition of MetS for Europeans [2,37]. Also, no association between the DII and MetS was reported in another Polish cross-sectional study conducted among inhabitants of Poland’s Świętokrzyskie Province and the city of Kielce, whose authors [18] observed an increased prevalence of abnormal WC among men in the DII quartile 4 (Q4) compared to those in quartile 1 (OR = 1.65; 95%CI = 1.01 to 2.69). Moreover, no association between the DII and MetS was reported in few European studies: the cross-sectional study “Observation of Cardiovascular Risk Factors in Luxembourg” (ORISCAV-LUX, *n* = 1352) [19] and the Spanish prospective study “Seguimiento Universidad de Navarra” (SUN) [23]. The ORISCAV-LUX study [19] found the association between proinflammatory DII scores (>1) and increased adjusted odds of having a low HDL-C. However, authors of the SUN study (median follow-up of 8.3 years, *n* = 6851), observed a significant association of a pro-vegetarian diet with a lower risk for developing MetS, but no significant association between DII and MetS [23].

In America, the authors of “The Buffalo Cardio-Metabolic Occupational Police Stress” (BCOPS, *n* = 464) found no correlation between a pro-inflammatory diet assessed by DII and MetS (OR for DII Q4 compared to Q1 = 0.87, 95% CI = 0.46–1.63) [38]. They reported a more prevalent glucose intolerance component among police officers in DII Q4 compared to Q1. Another study conducted on the Brazilian population (2017 adults) reported a mean DII score of 1.10, which indicates a pro-inflammatory diet, but no correlation between MetS and the DII score (men: prevalence ratio [PR], 0.98; 95% CI, 0.91–1.07; women: PR, 1.05; 95% CI, 0.91–1.20) [39].

In Asia, Ren et al. [40] reported a relatively limited association between the DII and the prevalence of MetS (with the exception of BP) among adults in eight cities in China. Similarly, in a study conducted among the Lebanese population (*n* = 336) [41] and in the Fasa Cohort Study (FACS) [42] conducted in Iran (*n* = 10,017), no significant association was reported between the DII and the prevalence of MetS.

However, there are a number of studies that have found a significant association between the DII score and MetS. In Europe, the authors of a French prospective study “the Supplémentation en Vitamines et Minéraux Antioxidants” (SU.VI.MAX, *n* = 3726) found an association between higher DII scores and a higher risk of developing MetS (OR for DII Q4 compared to Q1: 1.39; 95% CI = 1.01 to 1.92) [20]. In a 13-year follow-up, individuals in Q4 were more likely have higher systolic and diastolic BP, TG, and HDL-C [20]. A study conducted in the Irish population (Mitchelstown cohort, *n* = 3043) found an association of a pro-inflammatory diet with some MetS components, i.e., HDL-C, TG, FG, WC, and an overall association of MetS with higher DII scores (OR 1.37, 95% CI 1.01 to 1.88, *p* < 0.05) [22]. In the Croatian study, MetS prevalence of 25% was significantly associated with a pro-inflammatory diet as measured by DII [mean (SD) 3.28 (1.45); *p* < 0.01]. Multivariable logistic regression analysis showed a statistically positive association for a one-unit increase in the DII and MetS prevalence (OR = 2.31; 95% CI = 1.61–3.31), and elevated BP [21].

In America, the association between MetS and the DII was also observed in the cross-sectional US National Health and Nutrition Examination Survey (*n* = 17,689; OR for DII Q4 compared to Q1 = 1.65, 95% CI 1.44 to 1.89) whose authors found that the TG/HDL-C ratio, obesity, and high-sensitivity C-reactive protein (hsCRP) increased across DII quartiles [43]. The only exception was HDL-C levels, which decreased across DII quartiles. Two Mexican studies (*n* = 334 and *n* = 399) also confirmed the association of a pro-inflammatory diet with MetS [44,45]. Similarly, a study conducted in Latin America populations (*n* = 276) found a significant association between higher DII scores and MetS and/or cardiometabolic risk components: WC and diastolic BP [46].

In Asia, an association of pro-inflammatory outcomes evaluated by the DII score and MetS was observed in northern China [47], Iran [48,49], and Korea [50,51].

We observed a higher prevalence of increased TG across DII terciles (T1 vs. T3 OR 1.34 95% CI: 1.01–1.78), but after adjusting for confounding factors, this association was no longer statistically significant. Our logistic regression model was adjusted according to the criteria published by Qian Yi et al. [52] (BMI and physical activity, without the total energy intake). In addition, the model was adjusted for education, place of residence, smoking, and gender, which are important socioeconomic risk factors for CVD [53]. Increased risk of higher TG levels due to a more pro-inflammatory diet has also been shown in other studies [20,44,46,47,48]. In our study, a pro-inflammatory diet was characterized by a higher intake of red meat compared to the anti-inflammatory diet. Saturated fatty acids in processed red meat have been reported to activate a number of inflammatory pathways (mitogen-activated protein kinases (MAPK), nuclear factor kappa-B (NF-kB), and activator protein (AP)-1)), which may be associated with increased TG reserves in adipose tissue [54,55]. Similarly, the diets of participants in T3 had the highest intake of food products containing fructose, which is thought to induce lipid accumulation and hypertriglyceridemia, resulting in inflammation and hepatic steatosis [55,56]. Our study assessed the higher prevalence of abnormal FG in the anti-inflammatory diet (T1), which may be related to the cross-sectional nature of the study. Patients who were informed about their abnormal FG levels may have made some dietary changes before participating in the study, but the time was too short for these changes to be observed.

An anti-inflammatory diet (lower DII score) was characterized by a higher intake of vegetables, fruits, nuts, seeds, and fish. A pro-inflammatory diet, on the other hand, contained a lot of whole-fat foods, refined cereals, fats, fruit juices, red meat, processed meat, sugar-sweetened beverages, sweets, sugar, and honey [15,16]. Similar findings have been observed by other researchers comparing MetS and DII [22,44]. A so-called Western diet causes chronic inflammation and increases CVDs markers. Therefore, it is important to provide patients with appropriate dietary counseling to change their eating habits [15,16].

The cross-sectional nature of the study did not allow us to assess the causal association between the DII and MetS, which needs to be further confirmed in prospective studies. In addition, study participants were recruited through radio and television announcements, making the study sample not representative of the target population. The fact that this study was carried out with standardized methods and a validated high-quality FFQ, which included 154 food products and dishes specific to the Lower Silesian region, is a definite strength of the study. However, the above method in this study is limited because some DII components were not included in the questionnaire (saffron, eugenol, ginger, turmeric, pepper, rosemary, and thyme). This is the first cross-sectional study to determine the inflammatory potential of the diets of Poland’s Lower Silesia inhabitants, in which the DII was determined based on 37 food parameters. Also, due to the cross-sectional nature of the study design, study results corresponded to the actual dietary habits of study participants. No association between the DII and MetS reported in this study may be related to the fact that long-term risk factors for chronic diseases do not act until disease accumulates and manifests in the body. The real predictor is not the information whether disease occurs or not, but the exposure in a lifetime. Additionally, to get more accurate results, future studies should assess the role of inflammatory markers. The results of this study are only informative and provide an important basis for further research on the quality of diet and nutrition.

## 5. Conclusions

In conclusion, no association was found between the DII and MetS, except for increased TG concentrations in individuals in DII T3 compared to DII T1. However, this association was significant only in the crude model. Besides, our conclusion may be limited by the cross-sectional nature of the study. Therefore, more cohort studies are needed to fully understand whether diets with pro-inflammatory potential are associated with an increased risk of MetS and, possibly, with its specific components. Results of this study are informative and provide an important basis for further research on the quality of diet and nutrition.

## Figures and Tables

**Table 1:** Characteristics of food groups [30].

No.	Food Groups	FFQ Dietary Products
1.	Low-fat dairy products	Milk 1–2% fat, Buttermilk 0.5% fat Cocoa w/ milk 1–2% fat, Cottage cheese, Low-fat yoghurt, Kefir
2.	Full-fat dairy products	Milk 3.2% fat, Milk 3.2% fat (from mixed dish—oatmeal w/ milk), Feta Greek cheese Granulated cottage cheese w/ sour cream, Cheese, Edam cheese, Fromage cheese, Yoghurt 2–8% fat, Sour cream 12% fat, Sour cream 18% fat Sour cream 18% fat (from mixed dish—salad w/sour cream)
3.	Whole grains	Whole-meal rye bread, Mixed bread w/ rye and wheat flour w/ sunflower seeds, Buckwheat groats, boiled, Barley groats, boiled, Pasta w/ durum, boiled, Oatmeal (from mixed dish—oatmeal w/milk)
4.	Refined grains	Wheat bread, white, White rice, boiled, Wheat roll, white, Mixed bread w/ rye and wheat flour, white, Cornflakes
5.	Fats w/o oils	Butter, Lard, Fat spread w/ butter, Mayonnaise, Margarine, soft
6.	Raw fruit	Apple, Banana, Grapefruit, Grapes, Mandarin, Strawberries, Kiwi fruit, Lemon, Orange, Pear, Peach, Prunes, Raspberries
7.	Fruit juices	Orange juice; Raspberry juice; Carrot juice; Apple juice; Grapefruit juice; Black currant juice; Multifruit juice (local fruits); Multifruit juice (exotic fruits)
8.	Raw vegetables	Cabbage red, raw; Chinese cabbage, raw; Cabbage white, raw; Carrot, raw Cauliflower, raw; Chives; Cucumber, raw; Garlic cloves, raw; Salad, leaves; Onion, raw Parsley, leaves; Horseradish; Red pepper, raw; Radish; Tomato, raw; Sauerkraut salad Chinese cabbage salad w/ mayonnaise; Salad (from mixed dish—salad w/ sour cream)
9.	Cooked vegetables	Kidney beans, cooked; Beetroot, cooked; Broccoli; Cabbage white, cooked; Carrot, cooked; Cauliflower, cooked, w/butter; Mushrooms, fried; Red pepper, cooked Tomatoes, cooked; Tomato passata; Spinach, cooked; Zucchini, cooked Green beans, cooked; Corn, canned; Peas, canned; Salad of mixed cooked vegetables w/ mayonnaise
10.	Potatoes	Potatoes, boiled; Potatoes, mashed; French fries
11.	Lean meat	Chicken w/ skin boiled/fried; Chicken w/o skin boiled/fried Turkey, roasted
12.	Red meat	Beef, cutlets; Beef ham, boiled; Pork, bacon; Cutlets of ground beef and pork, fried; Offal
13.	Meat w/ breadcrumbs	Chicken nuggets; Pork chops, w/ breadcrumbs
14.	Processed meat/charcuterie	Chicken ham; Sausage; Luncheon meat, pork; Pork ham; Sausage, pork, smoked (traditional polish); Sausage, mixed beef/pork, smoked (traditional polish); Sausage, pork, white, boiled (traditional polish); Turkey ham; Turkey sausage ham; Brawn; Chicken pâté
15.	Eggs	Eggs, boiled/fried
16.	Fish	Codfish, fried, w/ breadcrumbs; Herring, w/ cream; Mackerel, smoked
17.	Mixed dishes	Baked beans w/ tomato sauce; Meat and rice stuffed cabbage w/ tomato Sauce; Dumplings w/ meat, boiled; Dumplings w/ potatoes and cottage cheese, Boiled; Sauerkraut and meat stew
18.	Beverages w/ added sugar	Fruit drink; Soft drink w/ added sugar
19.	Low-calorie beverages	Low-calorie soft drink
20.	Tea, coffee	Coffee; Tea, black; Tea, green/herb
21.	Alcohol	Beer; Wine; Vodka
22.	Sweets	Milk chocolate; Dark chocolate; Tea biscuit; Yeast cake; Shortbread cake; Gingerbread; Pound cake; Cheesecake; Halvah; Caramel candy; Other sweets; Candy; Ice cream
23.	Honey and sugar	Honey; Sugar
24.	Dried fruits	Raisins
25.	Nuts and seeds	Walnuts; Other nuts; Seeds
26.	Soups	Broth; Sour rye soup; Vegetable soup; Barley soup; Tomato soup; Bean soup; Sauerkraut soup

w/, with; w/o, without.

**Table 2:** Characteristics of study group by sex and total.

	Total *n* = 1570	Females *n* = 1000	Males *n* = 570	*p* *
Age, years, mean ± SD	54.65 ± 9.83	54.90 ± 9.74	54.22 ± 9.99	0.193
BMI, [kg/m^2^] mean ± SD	28.17 ± 5.15	27.84 ± 5.39	28.75 ± 4.66	0.001
Place of living, *n* (%)				
Rural	685 (43.6)	443 (44.3)	242 (42.5)	0.512
Urban	885 (56.4)	557 (55.7)	328 (57.5)	
Marital status, *n* (%)				
Married / living together	1166 (74.3)	667 (66.7)	499 (87.5)	<0.001
Never married	112 (7.1)	75 (7.5)	37 (6.5)	
Separated / divorced / widowed	291 (18.5)	258 (25.8)	33 (5.8)	
Education, *n* (%)				
Primary/trade	499 (31.8)	305 (30.5)	194 (34.0)	0.034
Secondary and high secondary	612 (39.0)	414 (41.4)	198 (34.7)	
University	459 (29.2)	281 (28.1)	178 (31.2)	
Smoking, *n* (%)				
Currently Uses Tobacco Products	332 (21.1)	200 (20.0)	132 (23.2)	<0.001
Formerly Used Tobacco Products	490 (31.2)	253 (25.3)	237 (41.6)	
Never Used Tobacco Products	748 (47.6)	547 (54.7)	201 (35.3)	
Alcohol, *n* (%)				
Currently use alcohol products	1081 (68.9)	633 (63.3)	448 (78.6)	<0.001
Formerly used alcohol products	162 (10.3)	98 (9.8)	64 (11.2)	
Never used alcohol products	327 (20.8)	269 (26.9)	58 (10.2)	
Physical activity, *n* (%)				
Low and moderate	418 (26.6)	254 (25.4)	164 (28.8)	0.163
High	1152 (73.4)	746 (74.6)	406 (71.2)	
DII, mean ± SD	−0.11 ± 2.91	−0.61 ± 2.88	0.77 ± 2.75	<0.001
DII, (min; max)	−7.88 to 7.33	−7.88 to 6.70	−6.75 to 7.33	
Metabolic syndrome, *n* (%)	664 (42.3)	401 (40.1)	236 (46,1)	0.023
Waist Component, *n* (%)	1094 (69.7)	698 (69.8)	396 (69.5)	0.938
BP Component, *n* (%)	1218 (77.6)	714 (71.4)	504 (88.4)	<0.001
FG Component, *n* (%)	614 (39.1)	379 (37.9)	235 (41.2)	0.213
TG Component, *n* (%)	387 (24.6)	212 (21.2)	175 (30.7)	<0.001
HDL Component, *n* (%)	310 (19.7)	234 (23.4)	76 (13.3)	<0.001

Groups compared with chi-square test for nominal variables and with *t*-test for continuous variables. * Statistical difference between groups of females and males. A *p*-value of <0.05 is considered statistically significant. Waist Circumference of ≥94 cm for males or ≥80 for females. A blood pressure (BP) component of >130 is systolic or >85 is diastolic. High-density lipoprotein (HDL-C) is <40 mg/dL in men and <50 in women. The triglyceride (TG) component is >149 mg/dL. The fasting glucose (FG) component is >99 mg/dL.

**Table 3:** Comparison of diet parameters (g/day) between DII terciles in total study group.

No.	Parameter	Total Group	Tercile 1	Tercile 2	Tercile 3	*p*	Post-Hoc
1.	Low-fat dairy Products	81.36 (39.22; 178.58)	83.50 (40.00; 192.58)	86.43 (37.07; 209.70)	74.93 (42.14; 142.82)	0.145	
2.	Full-fat dairy products	73.35 (38.22; 162.15)	46.02 (25.71; 83.21)	67.85 (39.87; 140.04)	150.00 (71.14; 284.98)	<0.001	1 < 2 < 3
3.	Whole grains	48.53 (30.00; 87.43)	51.69 (29.98; 93.53)	49.70 (29.65; 91.42)	46.17 (30.67; 73.55)	0.097	
4.	Refined grains	73.40 (21.08; 118.80)	25.71 (9.83; 64.29)	72.86 (27.27; 107.24)	110.71 (76.97; 155.71)	<0.001	1 < 2 < 3
5.	Fats w/o oils	17.81 (10.62; 29.75)	12.14 (6.43; 17.58)	17.30 (11.04; 25.36)	35.85 (18.94; 47.35)	<0.001	1 < 2 < 3
6.	Raw fruit	241.84 (160.29; 398.04)	266.85 (188.59; 448.69)	249.15 (157.10; 397.84)	217.14 (142.73; 324.01)	<0.001	1 > 2 > 3
7.	Fruit juices	117.68 (49.18; 214.29)	98.36 (32.79; 159.25)	114.75 (49.18; 194.96)	153.40 (68.50; 266.39)	<0.001	1 < 2 < 3
8.	Raw vegetables	134.92 (91.54; 197.90)	169.75 (111.21; 242.01)	128.49 (84.49; 191.57)	122.45 (82.84; 157.30)	<0.001	1 > 2 > 3
9.	Cooked vegetables	117.80 (79.75; 162.60)	125.62 (85.98; 184.93)	115.27 (76.44; 167.00)	112.02 (78.74; 147.77)	<0.001	1 > 2.3
10.	Potatoes	88.77 ± 57.66	75.58 ± 54.92	94.66 ± 58.60	96.06 ± 57.23	<0.001	1 < 2.3
11.	Lean meat	15.08 (13.11; 28.57)	14.29 (10.84; 28.57)	15.08 (13.11; 28.57)	15.08 (14.29; 22.81)	0.102	
12.	Red meat	18.57 (10.84; 28.59)	15.08 (8.52; 24.19)	17.70 (10.84; 28.59)	22.04 (13.47; 31.69)	<0.001	1 < 2 < 3
13.	Meat w/ breadcrumbs	20.84 (13.11; 28.57)	14.29 (6.56; 20.84)	20.84 (13.11; 28.57)	20.84 (13.11; 28.57)	<0.001	1 < 2.3
14.	Processed meat/charcuterie	50.07 (29.39; 89.33)	36.27 (20.32; 55.75)	46.24 (30.48; 80.56)	86.08 (42.61; 127.94)	<0.001	1 < 2 < 3
15.	Eggs	19.29 (6.43; 19.29)	6.43 (2.95; 19.29)	19.29 (6.43; 19.29)	19.29 (6.43; 19.29)	<0.001	1 < 2.3
16.	Fish	13.11 (6.56; 20.84)	9.84 (6.56; 16.98)	13.11 (6.56; 17.56)	14.29 (9.84; 20.84)	<0.001	1.2 < 3
17.	Mixed dishes	26.23 (13.11; 33.96)	19.67 (6.56; 28.57)	26.23 (14.29; 33.96)	26.23 (19.67; 33.96)	<0.001	1 < 2.3
18.	Beverages w/ added sugar	14.29 (6.56; 42.27)	6.56 (0.00; 22.95)	15.34 (6.56; 42.27)	16.39 (6.56; 50.00)	<0.001	1 < 2 < 3
19.	Low-calorie beverages	0.00 (0.00; 35.71)	0.00 (0.00; 250.00)	0.00 (0.00; 35.71)	0.00 (0.00; 0.00)	<0.001	1 > 2 > 3
20.	Tea, coffee	952.58 ± 459.02	1 081.96 ± 509.61	903.61 ± 438.31	872.26 ± 394.32	<0.001	1 > 2.3
21.	Alcohol	9.31 (0.00; 47.14)	9.31 (0.00; 47.14)	9.31 (0.00; 42.63)	12.13 (0.00; 49.96)	0.354	
22.	Sweets	41.48 (23.13; 63.36)	26.09 (13.44; 43.01)	40.76 (25.65; 61.41)	58.39 (40.43; 82.28)	<0.001	1 < 2 < 3
23.	Honey and sugar	15.43 (2.86; 21.14)	6.86 (1.31; 16.00)	16.00 (3.91; 22.29)	17.31 (9.71; 40.00)	<0.001	1 < 2 < 3
24.	Dried fruits	4.92 (0.00; 4.92)	4.92 (0.00; 4.92)	4.92 (0.00; 4.92)	4.92 (0.00; 4.92)	0.073	
25.	Nuts and seeds	5.44 (0.00; 9.00)	6.10 (1.43; 11.88)	5.44 (0.52; 9.33)	1.95 (0.00; 6.10)	<0.001	1 > 2 > 3
26.	Soups	223.89 (162.06; 307.26)	214.52 (157.38; 307.25)	228.57 (162.06; 317.92)	245.43 (183.61; 281.03)	0.026	1 < 2.3

w/, with; w/o, without; DII: Dietary Inflammatory Index. Data presented as mean ± SD or median (T1;T3), depending on data distribution. Tercile groups compared with ANOVA analysis or Kruskall-Wallis test. For ANOVA—a post-hoc Tukey test was applied, and for Kruskall-Wallis test—a post-hoc Dunn test was applied.

**Table 4:** Logistic regression odds ratio of metabolic syndrome and its components by DII terciles.

DII Tercile	Present *n* (%)	Absent *n* (%)	OR (95% Cl)	Adjusted OR (95% Cl) ^1^
Metabolic syndrome				
1	216 (32.5)	307 (33.9)	Ref.	Ref.
2	218 (32.8)	306 (33.8)	1.01 (0.79 to 1.29)	0.92 (0.69 to 1.23)
3	230 (34.6)	293 (32.3)	1.12 (0.87 to 1.43)	0.77 (0.56 to 1.06)
Raised WC				
1	362 (33.1)	161 (33.8)	Ref.	Ref.
2	356 (32.5)	168 (35.3)	0.94 (0.73 to 1.22)	1.02 (0.68 to 1.54)
3	376 (34.4)	147 (30.9)	1.14 (0.87 to 1.49)	1.22 (0.79 to 1.90)
Raised BP				
1	416 (34.2)	107 (30.4)	Ref.	Ref.
2	394 (32.3)	130 (36.9)	0.78 (0.58 to 1.04)	0.83 (0.59 to 1.15)
3	408 (33.5)	115 (32.7)	0.91 (0.68 to 1.23)	0.90 (0.63 to 1.29)
Reduced HDL-C				
1	95 (30.6)	428 (34.0)	Ref.	Ref.
2	113 (36.5)	411 (32.6)	1.24 (0.91 to 1.68)	1.27 (0.91 to 1.77)
3	102 (32.9)	421 (33.4)	1.09 (0.80 to 1.49)	1.02 (0.71 to 1.47)
Raised TG				
1	113 (29.2)	410 (34.7)	Ref.	Ref.
2	133 (34.4)	391 (33.1)	1.23 (0.93 to 1.65)	1.14 (0.83 to 1.55)
3	141 (36.4)	382 (32.3)	1.34 (1.01 to 1.78)	1.01 (0.73 to 1.41)
Raised FG				
1	204 (33.2)	319 (33.4)	Ref.	Ref.
2	186 (30.3)	338 (35.4)	0.86 (0.67 to 1.11)	**0.71 (0.54 to 0.94)**
3	224 (36.5)	299 (31.3)	1.17 (0.92 to 1.50)	0.78 (0.58 to 1.05)

DII: Dietary Inflammatory Index; OR: odds ratio; CI: confidence interval; Ref.: reference; BP, blood pressure; FG, fasting glucose; HDL-C, high-density lipoprotein cholesterol; TG, triglycerides; WC, waist circumference. ^1^ logistic regression models adjusted for sex, age, BMI, place of residence, education level, physical activity level, and smoking.

## Data Availability

Not applicable.
